# The ion implantation-induced properties of one-dimensional nanomaterials

**DOI:** 10.1186/1556-276X-8-175

**Published:** 2013-04-17

**Authors:** Wen Qing Li, Xiang Heng Xiao, Andrey L Stepanov, Zhi Gao Dai, Wei Wu, Guang Xu Cai, Feng Ren, Chang Zhong Jiang

**Affiliations:** 1 Department of Physics and Key Laboratory of Artificial Micro- and Nano-structures of Ministry of Education, Wuhan University, Wuhan, 430072, People's Republic of China; 2 Center for Electron Microscopy and Hubei Nuclear Solid Physics Key Laboratory, Wuhan University, Wuhan, 430072, People's Republic of China; 3 Kazan Physical-Technical Institute, Russian Academy of Sciences, Kazan, Republic of Tatarstan, 420029, Russian Federation

**Keywords:** Nanomaterials, Ion implantation, Doping

## Abstract

Nowadays, ion implantation is an extensively used technique for material modification. Using this method, we can tailor the properties of target materials, including morphological, mechanical, electronic, and optical properties. All of these modifications impel nanomaterials to be a more useful application to fabricate more high-performance nanomaterial-based devices. Ion implantation is an accurate and controlled doping method for one-dimensional nanomaterials. In this article, we review recent research on ion implantation-induced effects in one-dimensional nanostructure, such as nanowires, nanotubes, and nanobelts. In addition, the optical property of single cadmium sulfide nanobelt implanted by N^+^ ions has been researched.

## Review

### Introduction

One-dimensional nanomaterials have been reported plentifully, owing to its fascinating characteristics. One-dimensional nanomaterials, as an important member of the nanomaterial family, have been widely applied in the formation of a nanodevice. In recent years, several research have reported on various one-dimensional nanomaterial-based nanodevices, including field effect transistors (FETs) [1-4], nanogenerators [5], and solar cells [6]. Compared with conventional devices, nanodevices based on one-dimensional nanomaterials have certain characteristics, including superspeed, superhigh frequency; high integration density; and low power consumption. These characteristics impel one-dimensional nanomaterial-based nanodevices to be a vast potential prospect for future development in nanoelectronics and optoelectronics. All of these embody the excellent properties of one-dimensional nanomaterials. As two-dimensional nanomaterials, thin film materials also have special properties like quantum effect and broadened bandgap. Compared with thin film materials, one-dimensional nanomaterials have a more obvious quantum effect, higher surface energy, and larger surface activity. Nanowires/nanotubes/nanobelts as quasi-one-dimensional nanostructure are ideal building blocks for nanoscale devices.

With the advent of modern times, higher performance devices are desired. In order to get more high-performance devices, the pivotal problem is how to get better quality materials. Generally, we dope some impurities into the materials as a solution. Traditional doping methods can be roughly divided into three classes: doping during growth, doping by diffusion, and ion implantation. Doping with few impurities into one-dimensional nanomaterials has been achieved already, but controllable and reproducible doping is still difficult to be achieved during growth. Ion implantation is an advanced technique that has been widely applied in material surface modification for nearly 30 years. As a method for industrial application, ion implantation is a controllable and rather exact manner. Compared with conventional doping method, the prominent advantage of ion implantation is that almost all elements can be used for implantation and it never draws into any other impurity elements. Lately, focus ion beam (FIB) system has been used to perform ion implantation process [7,8]. In this method, the position of ion implantation becomes steerable. In this letter, we review literatures on the application of ion implantation on one-dimensional nanomaterials. Finally, we report on our work on the photoluminescence (PL) emission property of single CdS nanobelt implanted by N^+^ ions. CdS nanobelts have been marked by Au markers. Furthermore, the PL emission spectrum of every marked CdS nanobelts has been recorded before ion implantation. The experiment was designed to study the PL emission variation of the same CdS nanobelt after ion implantation.

### The changes of morphology and structure

Damages induced by ion implantation in an irradiated material are very different; they are related to the ion species, energy, fluences, beam current, and target material. All of these factors may impact the amount and type of the produced damage. While at high fluences, nanowires (NWs) have been observed to be bent and even completely amorphous [9,10]. Under low implantation fluences, it will only create some isolated point defects like vacancies and interstitials. When ions are implanted into the material, collision cascade may occur during the implantation process. Furthermore, this effect may cause abundant defects; a single implanted ion can create tens of thousands of vacancies and interstitials in the target materials [11]. However, most of these damages can be removed instantaneously by dynamic annealing [12]. Generally speaking, the collision has three independent processes, including nuclear collision, electron collision, and charge exchange. Among of these, nuclear collision pertains to elastic collision, and the result is that abundant defects will be created. Electron collision refers to the collision between incident ions and electrons of the target material, and this collision process pertains to an inelastic collision process. During the electron collision process, electrons of target atoms will probably be excited. Another process is the charge exchange between incident ions and target atoms. During this process, incident ions transfer energy to target atoms or electrons of target atoms, and the incident ions will be stopped within the target after multiple impacts.

Another important phenomenon is the sputtering effect. This effect generally impacts the shape and morphology of nanomaterials [13]. During the implantation process, as the collision cascades, induced by incident ions, the atoms of the target material may get enough energy to be ejected out from the target material [14]. On this account, the surface region of the nanowire will be sputtered away. This sputtering effect will be enhanced at low-lying areas, and then the nanowires will become rougher [15].

**Figure 1:**
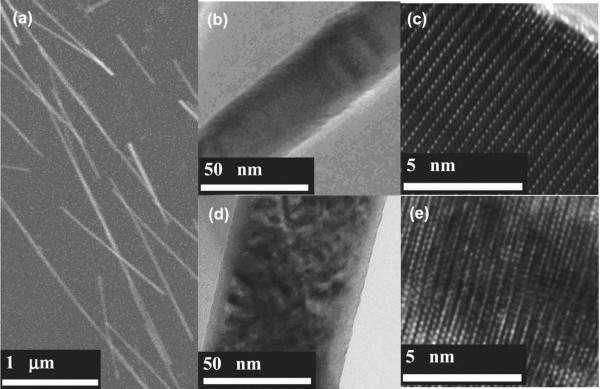
**SEM, TEM, and HREM images of ZnO nanowires.** (**a**) SEM image of ZnO nanowires dispersed on the substrate before ion implantation. (**b**) Low-magnification TEM image of the ZnO nanowire before ion implantation. (**c**) The corresponding high-resolution electron microscopy (HREM) image of nanowire in (**b**). (**d**) Low-magnification TEM image of ZnO after Er ion implantation (annealed). (**e**) The corresponding HREM image of nanowire in (**d**). Reprinted with permission from Wang et al. [16].

**Figure 2:**
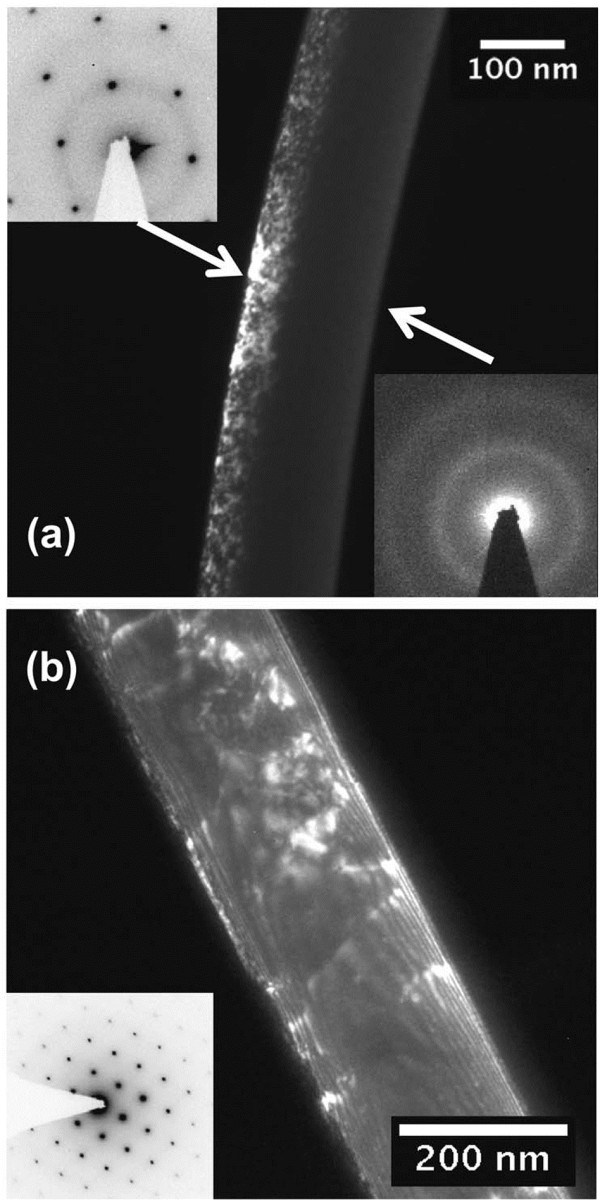
**Dark-field TEM images of GaAs nanowires after implantation and annealing.** (**a**) Zn implantation and (**b**) subsequent annealing at 800°C under arsenic overpressure. The insets in (**a**) show two corresponding diffraction patterns of selected areas, whereas the diffraction pattern in (**b**) is taken from the annealed nanowires. Reprinted with permission from Stichtenoth et al. [17].

Figure 1 shows the scanning electron microscopy (SEM) and transmission electron microscopy (TEM) images of the ZnO nanowires implanted by Er ions (reported by Wang et al.) [16]. Obviously, there are some deep recesses on the surface of the nanowire. In Figure 1e, it is apparent that the host lattice of the ZnO nanowire is repaired after annealing. Stichtenoth et al. [17] researched the Zn-implanted GaAs nanowires; they found that the right-hand side of the nanowire facing the ion beam incident direction had been amorphous, but the farther side was unimpaired. After annealing at 800°C for 30 min, the ion-implanted GaAs nanowire was fully re-crystallized; Figure 2b shows the dark-field image of the GaAs nanowire implanted by Zn ions and annealing at 800°C. Traditional annealing technologies include rapid thermal annealing and conventional furnace annealing. In general, the annealing temperature ordinarily keeps at two thirds of the melting point of the implanted materials [18]. Lately, Borschel et al. [19] reported that GaAs nanowires implanted by Mn^+^ at 250°C remained as single crystalline. However, polycrystalline nanowires were acquired after implantation at room temperature with subsequent annealing. It is noticeable that nanowires need higher implantation fluences to be amorphized compared with bulk materials; this is attributed to the enhanced dynamic annealing effect in nanowires.

**Figure 3:**
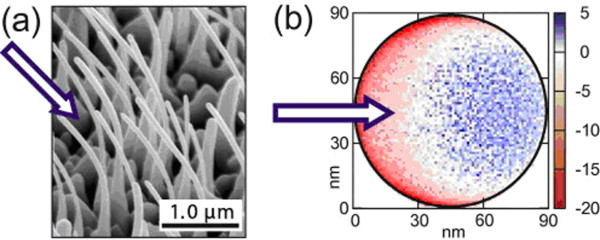
**SEM image of bent ZnO nanowires and result from *iradina *simulation**. (**a**) SEM image of bent ZnO nanowires after irradiation with 100 keV Ar ions. Arrow indicates ion beam direction. (**b**) Result from *iradina* simulation showing the distribution of damage within the nanowire. The different values of interstitials minus vacancies are shown (arbitrary units). Blue, excess interstitials; red, excess vacancies. Reprinted with permission from Borschel et al. [24].

What is more interesting is that the bending direction can be controlled by the ion species and implant energy [20,21]. In 2002, Kamins et al. [22] reported that the silicon nanowires bent away from the ion source after Ar^+^ ion implantation. Ronning et al. [23] explained this bending phenomenon as caused by defect accumulation. The nanowires bent away from the ion incident direction at low implant energy; in this situation, the damaged region was only the side of nanowires facing the incident direction. This effect may be attributed to the volume expansion of the nanowire part facing the incident direction. As the energy of the incident ions was low, the ions were only stopped within the side of the nanowires which is near the ion incident direction. In this circumstance, the nanowires got a heterogeneous volume expansion and then bent away from the incident direction. At larger implant energies, the nanowires bent toward the ion incident direction. In Figure 3, the arrows represent the ions incident direction (reported by Borschel et al.) [24]. In this case, most of the defects near the ion incident direction were vacancies, and the defects on the other side were almost interstitials. These two distinguishing patterns of defects led to an anisotropism expansion of the material. Figure 3b illustrates the simulation result of defect distribution. Furthermore, Jun et al. [10] reported a different phenomenon in Ga^+^ ion-implanted silicon nanowires with low implant energy (30 keV). They found that the silicon nanowires initially bent away from the ion beam and then bent toward the ion beam at higher doses; Romano et al. [25] also reported similar results. Park et al. [26] reported that the carbon nanotubes were bent using a FIB.

**Figure 4:**
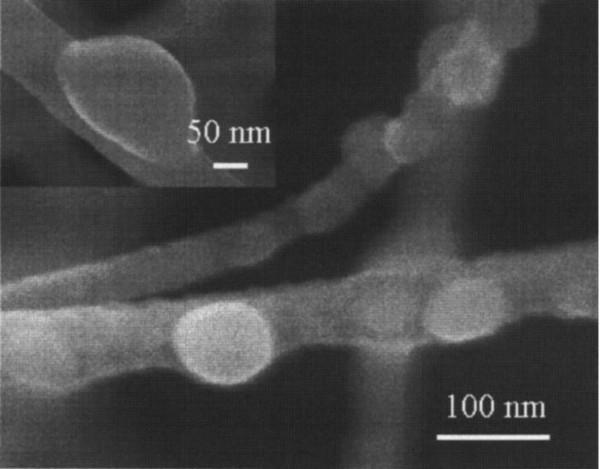
**FESEM images of bubbles formed at 50-keV Ga**^**+ **^**implantation on GaN nanowires.** The fluence was 2 × 10^20^ ions/m^2^. Inset shows a large bubble with a diameter of approximately 200 nm. Reprinted with permission from Dhara et al. [27].

Bubbles have been found in the film and bulk materials after ion implantation; afterward, this feature was also found in nanowires. Figure 4 shows the FESEM image of formed bubbles on the GaN nanowire which was caused by 50-keV Ga^+^ implantation (reported by Dhara et al.) [27]. Diameters of the bubbles are about 50 to 100 nm. The component of the bubbles is metallic α-Ga. The dominant mechanism for the generation of bubbles is the disintegration and accumulation of lattice atoms during implantation. As formation of nitrogen vacancies occurred, Ga atoms around nitrogen vacancies can also form a strong metallic bond.

Ion implantation not only causes the above-mentioned effects; Dhara et al. [28] reported that nanowires have a phase transformation after ion implantation. The Ga-implanted GaN nanowires transform from hexagonal phase to cubic phase. They ascribed this effect to two main reasons: one is that the accumulation of Ga ions have reduced the surface energy and stabilized the cubic phase, and the other possible reason is the short-range order fluctuations caused by dynamic annealing during the implantation process.

### The effect of the properties caused by ion implantation

When the ions are implanted into the nanomaterials, the ions will collide with the target atoms and charges. As noted previously, the collision processes include three different modes: nuclear collision, electron collision, and charge exchange. Incident ions lose the energy during every collision process and may be stopped within the materials as impurity atoms. It is common that most of these incident ions stay at the interstitial sites, and these interstitial impurities may migrate to substitutional positions after annealing. This substitutional doping enables the nanomaterials to get more admirable properties.

#### Electrical properties

After ion implantation and annealing, the carrier concentration of nanomaterials may increase dramatically and even the conductive type of nanomaterials may be converted by this fierce process. Without annealing, the implanted nanomaterials revealed worse conductivity, attributing to the damaged crystal lattice. In order to recover the crystal lattice, subsequent annealing is essential. On the other hand, annealing also provides the condition to activate impurity atoms.

**Figure 5:**
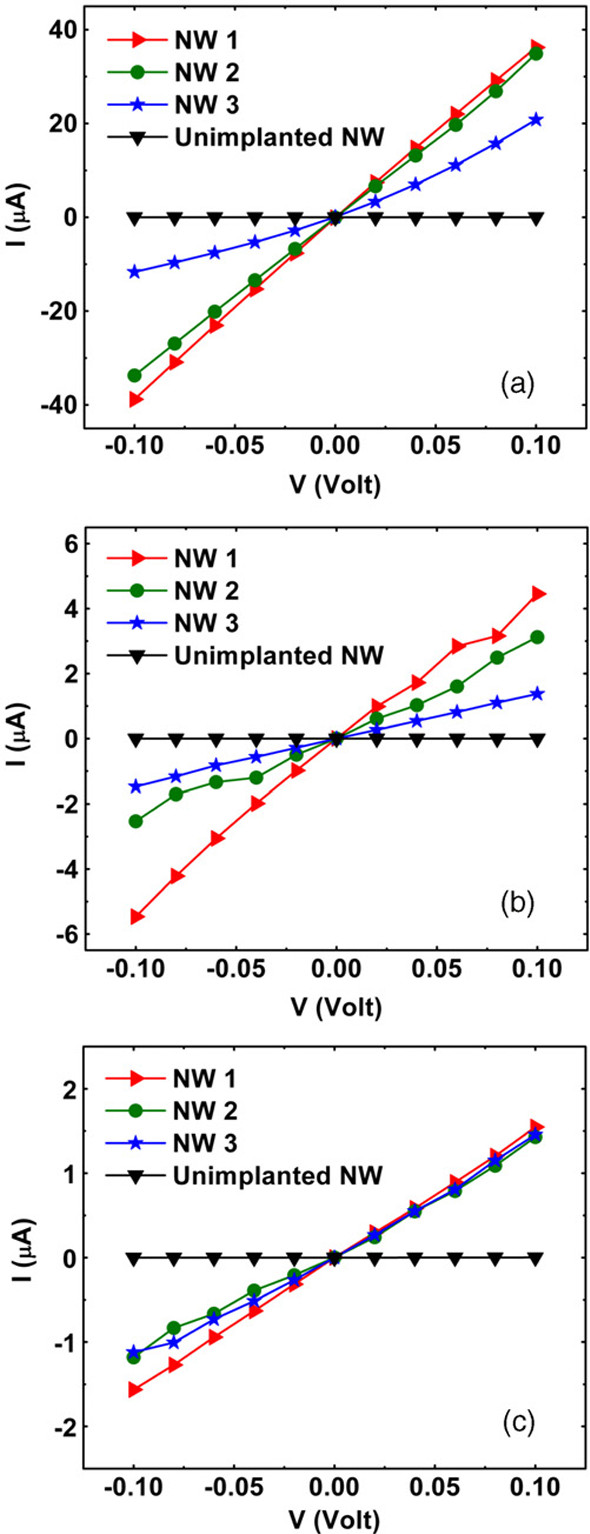
***I-V *curves of nanowires.** (**a**) three B-implanted NWs, (**b**) three P-implanted NWs and (**c**) three As-implanted NWs. *I-V* curve of an as-grown, unimplanted NW is included in each case for comparison. Reprinted with permission from Kanungo et al. [29].

**Figure 6:**
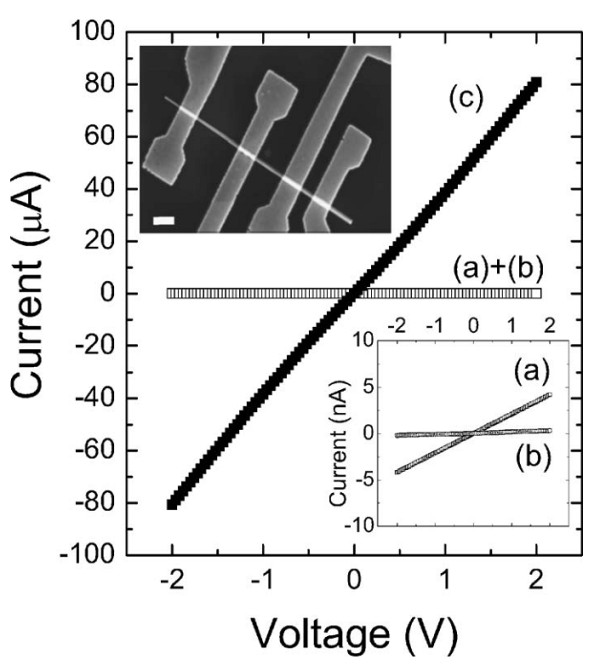
**Ohmic current–voltage characteristics of TLM structures.** These TLM structures (see inset scale bar 1 μm) are prepared on (**a**) as-grown, (**b**) as-grown and annealed, and (**c**) Zn-implanted and annealed GaAs nanowires. The second inset shows the *I*-*V* curves of (**a**) and (**b**) in a more adequate current scale. Reprinted with permission from Kanungo et al. [17].

Kanungo et al. [29] utilized ion implantation to achieve the n*-* and p*-*doping of silicon nanowires. Figure 5a,b,c shows the *I-V* curves of B-implanted Si nanowires, P-implanted Si nanowires, and As-implanted Si nanowires, respectively [29]. In all the *I-V* curves of the implanted nanowires in Figure 5, compared with those of the unimplanted nanowires, the conductivity of the implanted nanowires were observably enhanced. Comparing all the curves of Figure 5, the B-implanted Si nanowires have the highest conductivity. Boron is a light element which can easily substitute for the silicon ions at 850°C, and high-crystalline quality B-doped Si nanowires were acquired after subsequent annealing. P-implanted Si nanowires and As-implanted nanowires revealed lower conductivity; this must be attributed to the enhanced surface depletion [30]. The interaction of defects enhanced the diffusivity of the P atoms [31]. After annealing, most of the P atoms diffused out of the Si nanowires. These atoms staying on the surface of the nanowires can enhance the surface depletion. Stichtenoth et al. [17] fabricated p-type doped GaAs nanowires by zinc ion implantation. After Zn ion implantation, the sample was annealed at 800°C for 30 min, and then the conductivity of the GaAs nanowire increased in several orders of magnitude (Figure 6). Zeiner et al. [32] reported Ga-doped Ge nanowires fabricated through FIB. After Ga ion implantation, the conductivity of the Ge nanowires improved to approximately two orders of magnitude, but with implantation fluences above 6.25 × 10^12^ ions/cm^2^, the conductivity of the Ge nanowire fell sharply. In the paper, the author ascribed the increase in conductivity to a substitutional activation of Ga in the Ge nanowires; the conductivity decrease at high doses is attributed to defect generation and, finally, amorphization. Paschoal et al. [33] reported that the transport characteristic of Mn^+^-implanted GaAs nanowires is governed by nearest neighbor hopping at high temperature (*T* > 180 K) and Mott variable range hopping at low temperature (50 K <*T* < 180 K). Yan et al. [34] reported that conductivity of the carbon nanotube (CNT) networks is enhanced by H ion beam irradiation.

**Figure 7:**
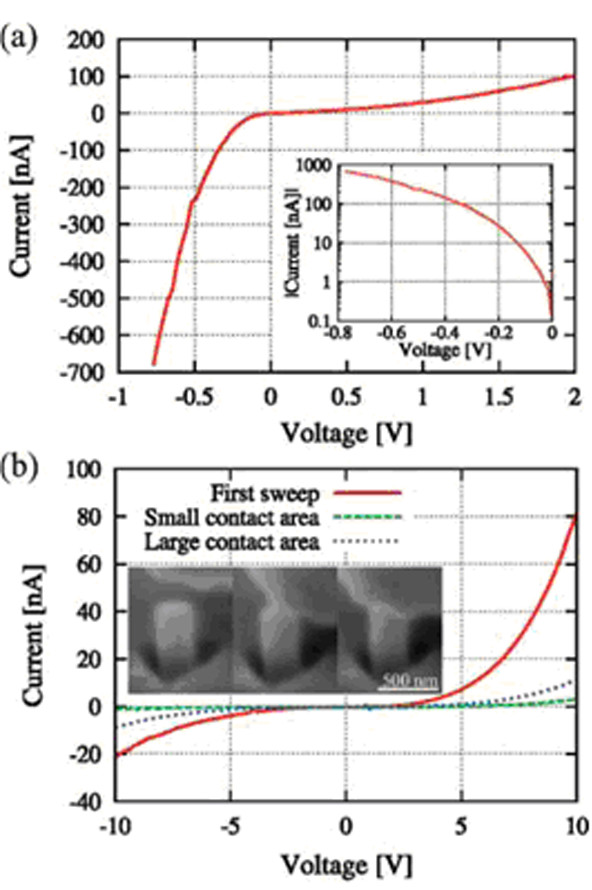
***I-V *curves of n-p and p-n nanowires.** (**a**) n-p Nanowires (n-doped at the top and p-doped at the bottom) and (**b**) p-n nanowires (p-doped at the top and n-doped at the bottom). Reprinted with permission from Hoffmann et al. [35].

The major aim of doping in nanowires is to produce a p-n junction in semiconductor nanowires. Hoffmann et al. [35] demonstrated a method to produce an axial p-n junction in silicon nanowires by ion implantation. By varying the implantation energy, the incident ions can stay at different sites in the nanowire. Hoffmann et al. implanted P and B ions into vertically aligned silicon nanowires to produce p-n junctions inside the silicon nanowire. Figure 7 shows the *I-V* curves of silicon nanowires which have already formed p-n junction by ion implantation. A typical *I-V* curve of the n-p junction is shown in Figure 7a. All the *I-V* curves in Figure 7b show a rectifying behavior, but the conductivity of the nanowires with different probe-nanowire contact type has a different magnitude. The red curve is the first recorded sweep (contact types show in the left inset). The phenomena that appeared in Figure 7b may be attributed to the Schottky barrier formed between the nanowire and the probe. Several months later, Kanungo et al. [36] reported another method to fabricate axial p-n junctions in silicon nanowires. They fabricated vertical silicon nanowires; the lower halves of the nanowires were doped with boron, and then phosphorus ions were implanted into the upper halves of the nanowires.

**Figure 8:**
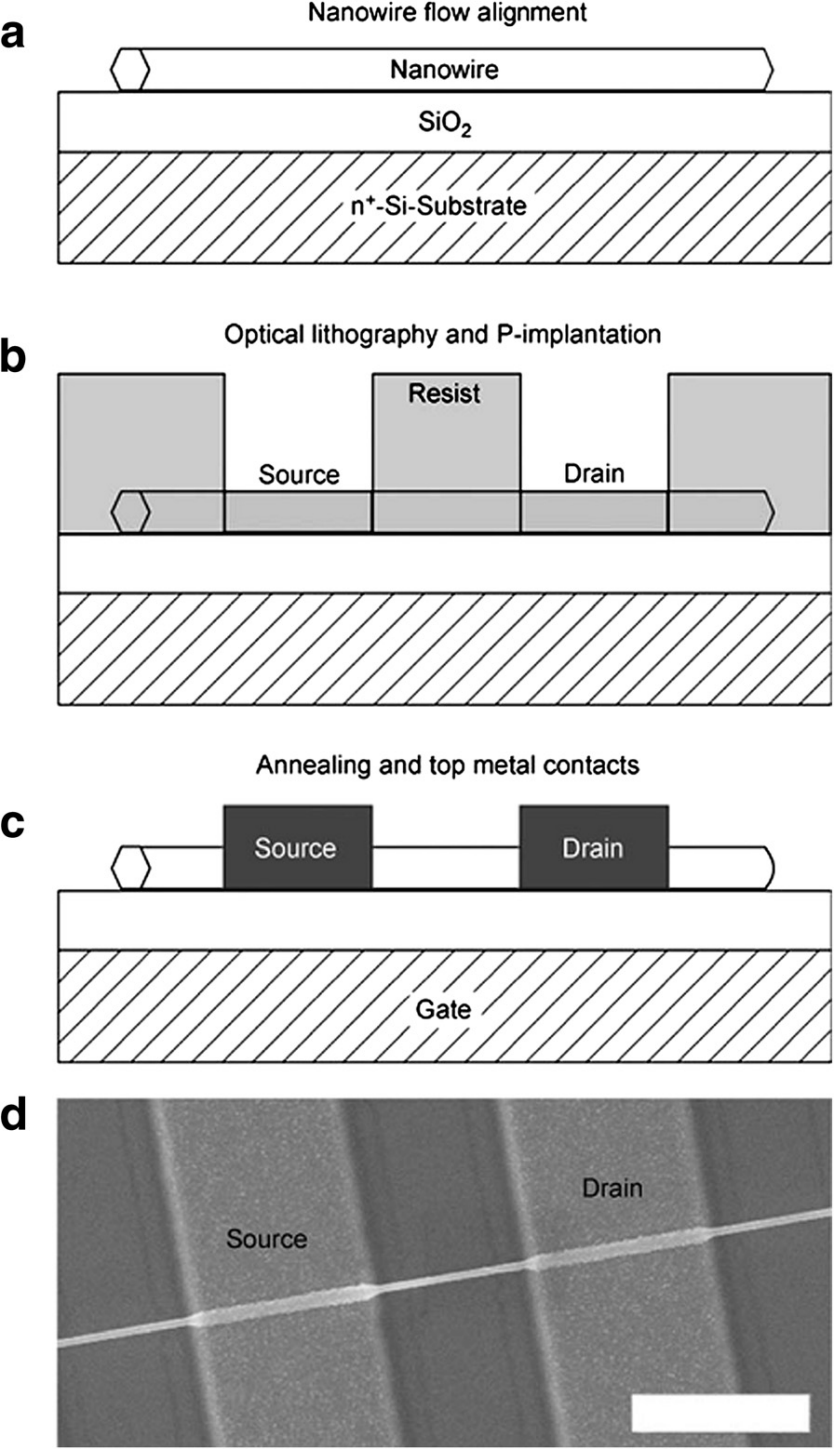
**Preparation process of nanowire devices.** (**a**–**c**) Schematic representation of the NWFET fabrication. (**d**) SEM micrograph of a nanowire device with top contacts. Reprinted with permission from Hayden et al. [37].

Optical lithography and e-beam lithography have been widely used in the formation of microelectronic devices, and these two technologies combined with ion implantation have been already applied to fabricate FET. Hayden et al. [37] utilized optical lithography and ion implantation to produce an n-type/intrinsic/n-type junction in the silicon nanowires. With the n-doped substrate under the silicon oxide layer as the global back gate, metal oxide semiconductor FET was finished by ion implantation and optical lithography (details in Figure 8). Colli et al. [2] implanted P or B ions into silicon nanowires that have a thick oxide shell surrounding the silicon core and then evaporated Ni on the silicon nanowires as the electrode through e-beam lithography. Throughout the entire experimental process, it is the crucial step to choose the appropriate implantation energy. It must be ensured that the dopants were stopped within the core of nanowires. The incident ion energy and implantation fluences may impact the quality of the FETs. Jang et al. [38] reported that the CNT-FET exhibited p-type behaviors after oxygen implantation at low doses and metallic behaviors at high doses. Zinc oxide nanowires have been widely applied in the fabrication of FETs; Liao et al. [39] utilized Ga^+^ ion implantation to improve the performance of nanowire-based FETs. The improvement of the performance is attributed to a reduced surface effect after ion implantation. There are many other semiconductors used to produce FET, but there is still little for doping through ion implantation.

#### Optical properties

Owing to the desirable optical properties of semiconductor nanomaterials, many nanomaterials were used to fabricate light-emitting diodes [40-42] and nanowire lasers [43]. However, there are still some imperfections of these nanodevices; doping with optically activated impurities (like transition metals and rare earth elements) through ion implantation may improve the properties of these nanodevices [44]. Transition metals (TM) are interesting doping elements for semiconductor nanowires because of its enormous optical influences to semiconductor nanowires. Doping with rare earth elements is another significant research direction, as rare earth elements have a special outermost electron structure [45].

**Figure 9:**
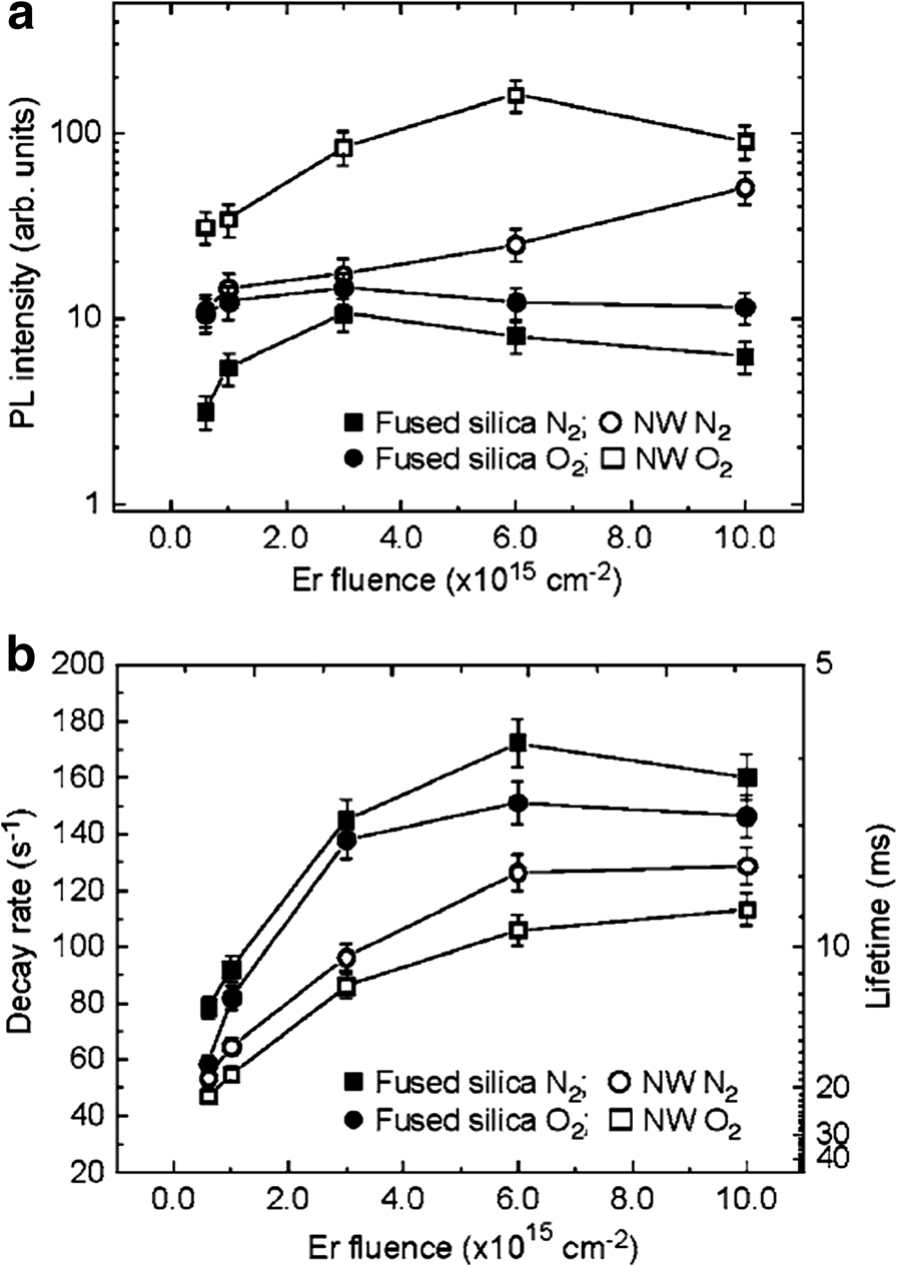
**Room-temperature PL measurements of bulk and NW samples as a function of ErO**^**- **^**implant fluence and ambience.** (**a**) Integrated PL intensity and (**b**) luminescence decay rate (lifetime). Reprinted with permission from Elliman et al. [46].

Silica nanowires are significant nanomaterials for integrated photonics and biosensing because silica nanowires are suitable hosts for optically active impurities, are chemically inert, and are excellently biocompatible. Elliman et al. [46] reported silica nanowire doping with erbium by ion implantation, and they found that luminous intensity and lifetime have a very obvious enhancement. Figure 9 shows the function relation of PL intensity and decay rate relative to the Er ion implantation fluence. All the testing processes have been performed at room temperature and subsequently annealed at 900°C in N_2_ and O_2_ to optically activate the erbium. Compared with bulk silica, the PL of silica nanowires reveals stronger intensity and longer lifetime. The PL intensity of bulk silica increased after ion implantation, but it decreased with the augmentation of implantation fluence. After ion implantation, the PL lifetime of the material decreased. This behavior is attributed to concentration quenching caused by ion implantation [47]. The concentration of nonradiative defects will increase during the implantation process. All samples annealed in O_2_ have stronger PL intensity and longer lifetime than the samples annealed in N_2_. Annealing in the O_2_ atmosphere increases the concentration of Er^3+^ and reduces the oxygen-deficient defect centers in silica. The PL intensity of the material is related to the Er^3+^ concentration, and the PL lifetime is related to the concentration of nonradiative defects.

**Figure 10:**
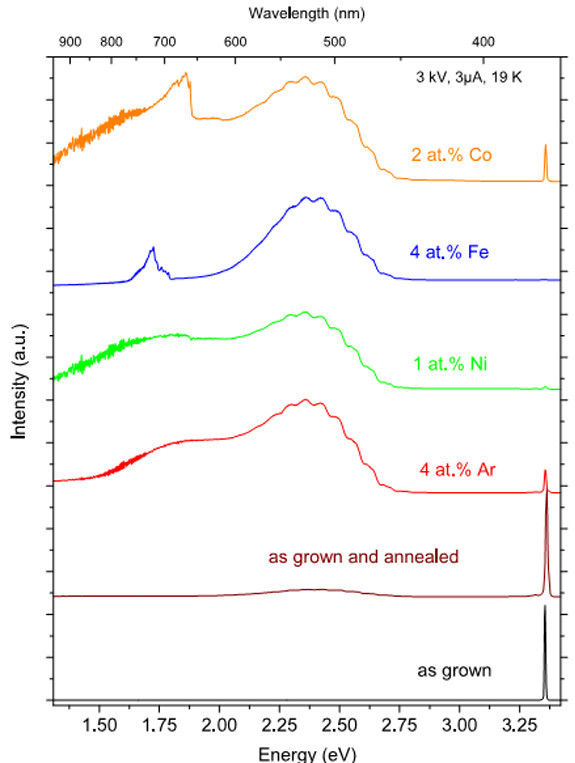
**Cathodoluminescence of Ni-, Fe-, Co- and Ar-implanted ZnO nanowire compared to as-grown ZnO nanowires.** Reprinted with permission from Müller et al. [49].

In recent years, short wavelength laser has been widely researched. ZnO has high optical gain and energy conversion efficiency excited by UV light at room temperature. The luminescence spectrum of ZnO has good monochromaticity. All these characteristics impel ZnO to be a tremendous prospect for optical device application. The ZnO NW-based optically pumped laser has already been realized by Zimmler et al. [48]. ZnO can realize multiband luminescence by doping with optically active elements; this property provides a possibility to fabricate various color optical devices. Müller et al. [49] researched the luminescence of transition metal-implanted ZnO nanowires. Figure 10 shows the cathodoluminescence spectra of Ni-, Fe-, Co- and Ar-implanted and as-grown ZnO nanowires. In Figure 10, the as-grown nanowire reveals a sharp UV luminescence. The cathodoluminescence of the ion-implanted nanowires is obviously different from that of the as-grown nanowire. After annealing, Ar diffused out of the lattice, and transition metal elements occupied the zinc lattice site [50,51]. The increasing interstitial zinc acts as a shallow donor. The concentration of the interstitial oxygen increased after annealing, and the interstitial oxygen is a deep acceptor. All the implanted samples show a structured green luminescence ascribing to the transition from the shallow donor to the deep acceptor. In the red luminescence region, Co- and Fe-implanted ZnO nanowires reveal an obvious intra-shell luminescence. Ronning et al. [52] reported the ZnO nanobelts implanted with 30-keV Mn^+^ ions; after annealing at 800°C, the structure and luminescence of ZnO nanobelts were recovered.

There are many other II-VI and III-V semiconductor nanomaterials that deserve to be researched like ZnS, GaN, ZnSe, and CdTe. One-dimensional nanomaterials have also been widely applied in the field of photocatalysis.

#### Magnetic properties

Several research about diluted magnetic semiconductor (DMS) have become much more attractive since Dietl et al. predicted that several wide bandgap semiconductors possibly have a room temperature Tc, including GaN and ZnO [53]. Low-dimensional DMS materials like nanowires have a significant application in spintronic nanodevices. The most important assignment is the synthesis of suitable DMS materials. Many papers reported that they can get room-temperature ferromagnetism through TM doping in the semiconductor materials, but some other researchers did not acquire room-temperature ferromagnetism through almost the same method. Ion implantation, as an effective doping method, plays an important role in the preparation of DMS.

**Figure 11:**
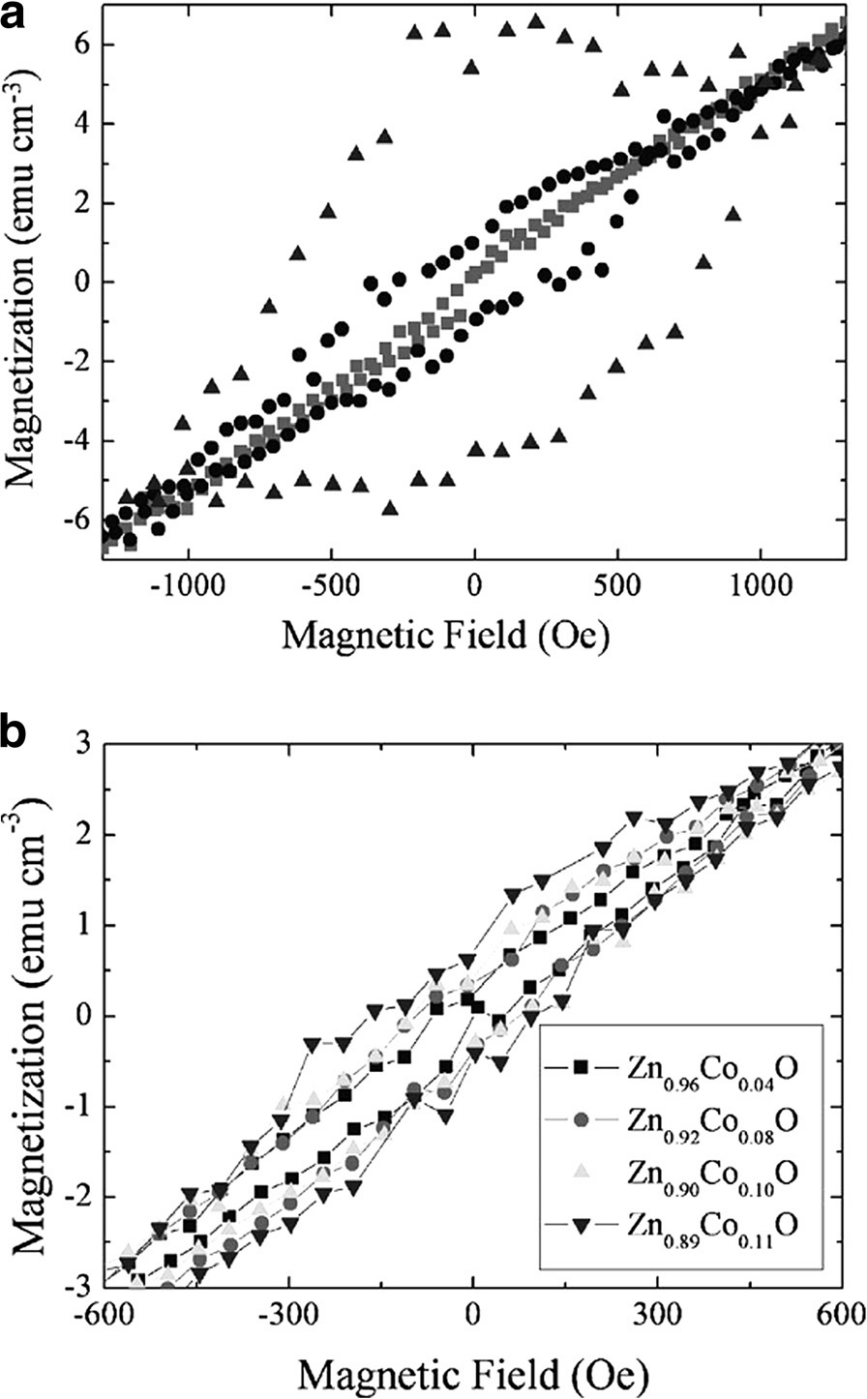
**Magnetization as a function of applied field at 2 K for Zn**_**0.89**_**Co**_**0.11**_**O and Zn**_**1-*x***_**Co**_***x***_**O NWs.** (**a**) Magnetization as a function of applied field at 2 K for as-implanted (squares), argon-annealed (circles), and vacuum-annealed (triangles) Zn_0.89_Co_0.11_O NWs. (**b**) Magnetization as a function of applied field at 2 K for argon-annealed Zn_1-*x*_Co_*x*_O NWs. Reprinted with permission from Jian et al. [58].

ZnO is the most fascinating II-VI semiconductor; room-temperature ferromagnetism of TM-doped ZnO has been reported [54,55]. However, some other research did not reveal any ferromagnetism signal [56,57]. There is also an argument about the origin of room-temperature ferromagnetism of these TM-doped materials. Jian et al. [58] reported that ferromagnetism of Co-implanted ZnO nanowires has a close connection with the structural order. In their work, the ZnO nanowire grew through thermal evaporation and then implanted by Co ions. In Figure 11a, the squares represent the as-implanted NWs, the circles represent the argon-annealed NWs, and the triangles represent vacuum-annealed NWs. After annealing, the implanted sample revealed an enhanced hysteresis loop, and as the annealing temperature increased, the hysteresis loop was squeezed. Jian, Wu et al. considered that it is related to the increased number of carriers; the theory on carrier-mediated ferromagnetism may explain this phenomenon [59]. Annealing was performed once again in oxygen and argon atmosphere for the already annealed sample under high vacuum. The results reveal that the hysteresis loop of the oxygen-annealed sample has decayed and the argon-annealed sample almost has no change. Annealing in oxygen may cause the reduction of oxygen vacancies and concentration of carriers. Figure 11b shows the *M-H* curves of different doping quantity of nanowires; the hysteresis loops increase with the increasing concentration of Co ions. Shuai et al. [60] reported that the Cu^+^-implanted ZnO nanowires have room-temperature ferromagnetism. The ZnO nanowires were implanted with 100-keV Cu^+^ ions and then annealed at 600°C for 2 h in argon and oxygen atmosphere. They found that the oxygen-annealed samples have stronger ferromagnetism than the argon-annealed samples.

**Figure 12:**
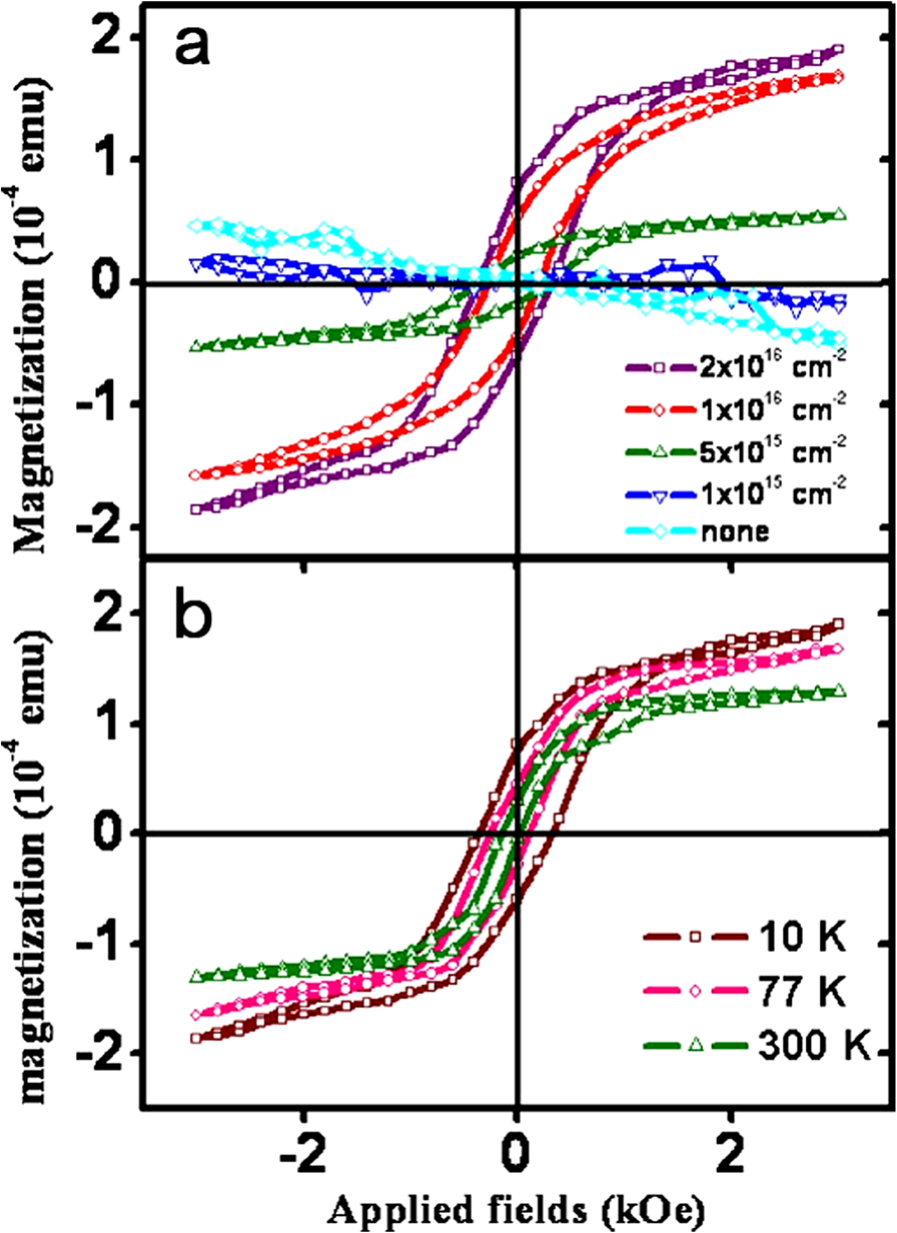
**Hysteresis loops measure at various temperatures.** Hysteresis loops (**a**) measured at 10 K for Si nanowires Mn^+^-implanted to doses of 1 × 10^15^, 5 × 10^15^, 1 × 10^16^, and 2 × 10^16^ cm^-2^ and (**b**) taken at 10, 77, and 300 K for Si nanowires Mn^+^-implanted to a dose of 2 × 10^16^ cm^-2^. Reprinted with permission from Wu et al. [61].

Wu et al. [61] reported on room-temperature ferromagnetism of Mn^+^-implanted Si nanowires. Figure 12 shows magnetization as a function of applied field for Si nanowires implanted with different fluences. Figure 12a shows that saturation magnetization increased with increasing Mn ion concentration. This phenomenon reveals that the magnetic moments' long-range ferromagnetic coupling is related to the Mn concentration. Figure 12b shows that the hysteresis loops and saturation magnetization increase with the reduction of temperature. Pure Si nanowires are diamagnetic, and all of the manganese silicide phases are not ferromagnetism. However, Mn-implanted Si nanowires reveal a room-temperature ferromagnetism that can be attributed to the long-range ferromagnetic coupling that occurred between electrons and Mn atoms.

GaAs [62] and GaN [63,64] as III-IV semiconductors have excellent properties to fabricate DMS; TM-implanted GaN has a high Tc (≧300 K) [53]. So far, the origin of room-temperature ferromagnetism of the TM-implanted DMS was not clear. The low repeatability of room-temperature ferromagnetic semiconductors is another problem.

### Nitrogen-implanted single cadmium sulfide nanobelt

**Figure 13:**
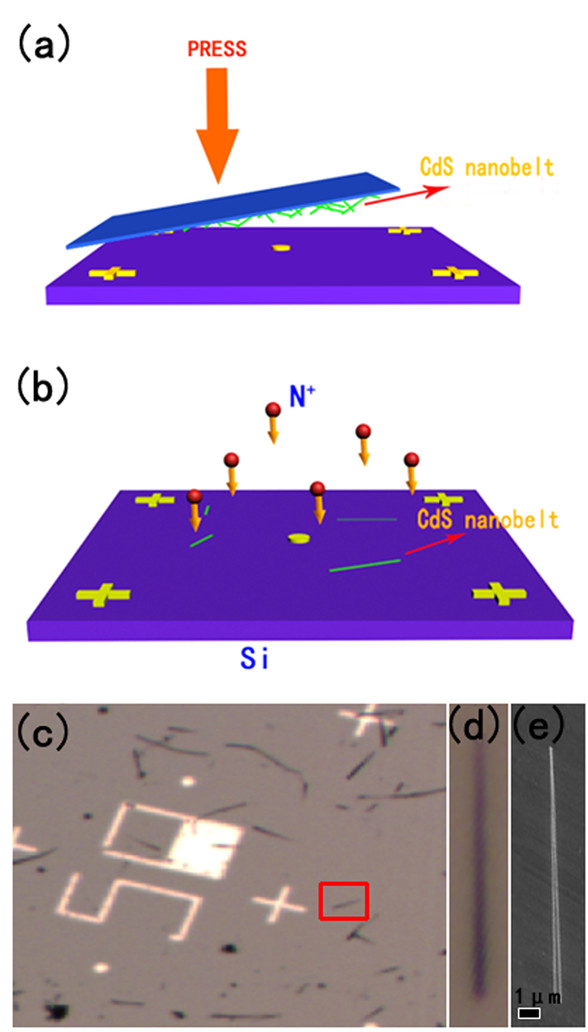
**Schematic diagram and optical and SEM images of processes.** The schematic diagram of (**a**) the transform process and (**b**) implantation process. (**c**, **d**) The optical and (**e**) SEM image of the nanobelts grown by thermal evaporation process.

Cadmium sulfide (or CdS) is a representative wide-bandgap II-VI semiconductor; its bandgap is 2.42 eV at room temperature. Cadmium sulfide has been extensively applied to fabricate optical cavities, waveguides, lasers, and solar cells. Many research on ion-implanted CdS film were reported substantially, and most of these research discussed the optical property of CdS films. In spite of this, papers reporting about CdS nanobelts were quite a few; ion-implanted single CdS nanobelts have seldom been researched. From this perspective, we studied the optical property of the N^+^ ion-implanted single CdS nanobelts and expected that the energy band structure of the CdS nanobelts could be transformed by ion implantation. Different from previous reports, the selected CdS nanobelts were marked by an Au marker; by this, it means that property variation process of the marked CdS nanobelts can be recorded. The CdS nanobelts were acquired by thermal evaporation process; the CdS powers were evaporated at 850°C in a tube furnace with Au as the catalyst on the silicon substrate. CdS nanobelts with 200- to 500-nm widths and tens of microns long were fabricated by this catalyst-assisted VLS process. Next, the nanobelts were transformed on another silicon chip, and Au markers had been produced on the silicon chip in advance through photolithography. The prepared samples were mounted into the vacuum chamber of the ion implanter and implanted by N^+^ ions with 30 keV. The choice implantation fluences include 5 × 10^15^, 1 × 10^16^, and 5 × 10^16^ ions/cm^2^. The photoluminescence spectra of every marked CdS nanobelts were detected by the micro-Raman system (LabRAM HR800, HORIBA Ltd., Minami-Ku, Kyoto, Japan) both before and after ion implantation. Surface morphology images of CdS nanobelts were acquired through SEM (FEI Sirion FEG, FEI Company, Hillsboro, OR, USA). Figure 13a,b shows schematic diagrams of the transfer process and implantation process, respectively. Figure 13c,d,e displays the SEM and optical image of the CdS nanobelts.

**Figure 14:**
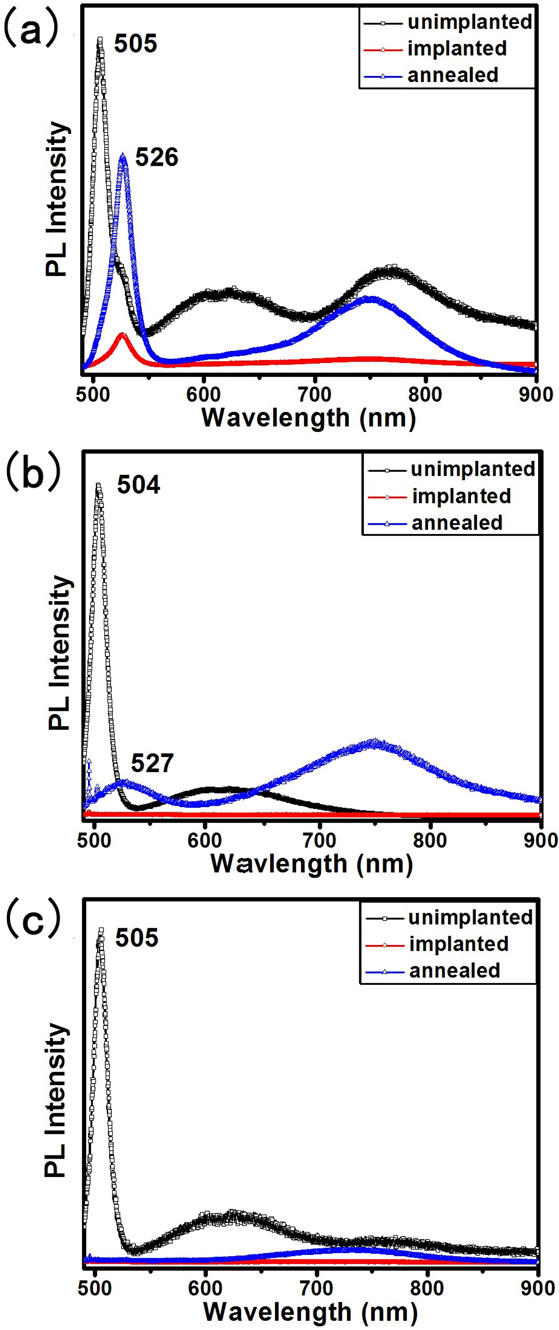
**PL emission spectrum of CdS nanobelts.** They are implanted by N^+^ ions with doses of (**a**) 5 × 10^15^, (**b)** 1 × 10^16^ and (**c**) 5 × 10^16^ ions/cm^2^.

Figure 14 shows the PL emission spectrum of single CdS nanobelts at room temperature. All the curves in Figure 14a signify the PL emission spectrum of the same nanobelt; Figure 14b,c represents two other nanobelts. In the case of the dose of 5 × 10^15^ ions/cm^2^, the PL emission spectrum of the unimplanted nanobelt has three emission peaks at about 505, 617, and 770 nm. The peak at 505 nm originates from the near-band-edge emission of CdS, and the broad emission band at 617 nm is associated with the low density of sulfur vacancies in the CdS nanobelt [65]. The peak at 770 nm is related to the transitions between the surface states and the valence band of CdS [66,67]. After ion implantation, the near-band-edge emission peak was red-shifted, and the defect emission peak was quenched. Later, all the samples were annealed in an argon atmosphere at 350°C for 40 min. The crystalline quality of the CdS nanobelts recovered obviously after annealing in argon atmosphere. In the red emission region, the annealed nanobelts have an emission peak at 750 nm. This may be attributed to the surface defect similar to that of unimplanted nanobelts and/or the high density of sulfur vacancies caused by ion implantation [65,68]. Unimplanted nanobelts have a defect emission peak at 617 nm caused by a small number of sulfur vacancies generated during growth process. After ion implantation and the annealing process, the concentration of sulfur vacancies increased observably; although the annealing process could recover the crystal lattice and reduce sulfur vacancies, a mass of sulfur vacancies still remained in the lattice after annealing. The emission peak at 526 nm may be attribute to the N^+^ ions implanted into the crystal lattice and substituted S as a shallow acceptor; this process resulted in the red shift of the band-edge emission peak. Figure 14b,c displays the PL emission of the CdS nanobelts implanted by N^+^ ions with the dose of 1 × 10^16^ and 5 × 10^16^ ions/cm^2^, respectively. When the implantation fluence increased to 1 × 10^16^ ions/cm^2^, the CdS nanobelts almost became amorphous and the photoluminescence were quenched. After annealing at 350°C, the crystal lattice recovered and PL emission peaks reappeared, such as that which occurred in the situation in the dose of 5 × 10^15^ ions/cm^2^, whereas the crystal lattice did not recover after annealing in the case of 5 × 10^16^ ions/cm^2^ (Figure 14c) which may be attributed to the CdS nanobelts being seriously damaged by implantation process.

## Conclusions

Many growth methods have been used to fabricate nanowires; with the development of technology, growth methods become outmoded, and various kinds of nanomaterials are developed. These nanomaterials have been applied in fabricating high-performance electronic or optical devices. With the purpose of getting higher performance devices, various elements were doped into the nanomaterials. Nevertheless, doping is not effortless; p-type doping of certain materials, such as CdS and ZnO, are rather knotty. Obviously, ion implantation is the most accurate and controllable method for doping, and theoretically, ion implantation can be appropriate for almost all the elements. We need not consider solubility limits and never fear to introduce impurity elements. After ion implantation, the electrical conductivity of nanowires can be increased by several orders of magnitude. The p-n junctions can be created in vertically grown nanowires after ion implantation. Ion implantation has also been utilized to fabricate nanoscale electrical devices. Implanted nanowires show a different optical characteristic compared to the as-grown nanowires. After ion implantation, the luminescence spectrum of the nanowires may be broadened and the bandgap will be changed. These properties changed by ion implantation are important in fabricating optical devices. Research on diluted magnetic semiconductor nanowires still has a long way to explore. The origin of room-temperature ferromagnetism should be figured out.

With technological improvements, devices inch toward the mini size; in this situation, accurate doping of nanomaterials becomes significant. Consequently, accurate and effective doping of one-dimensional nanomaterials will be the focus of research. We will focus on this field in the future.

## Abbreviations

CNT: Carbon nanotube; DMS: Diluted magnetic semiconductor; FET: Field effect transistor; FIB: Focus ion beam; NWs: Nanowires; PL: Photoluminescence; TM: Transition metal.

## Competing interests

The authors declare that they have no competing interests.

## Authors’ contributions

WQL participated in material preparation and data analysis and drafted the manuscript. XHX conceived and co-wrote the paper. ALS, FR, WW, GXC, and ZGD participated in the sample characterization. CZJ participated in its design and coordination. All authors read and approved the final manuscript.
